# Evaluation of Advanced Nanomaterials for Cancer Diagnosis and Treatment

**DOI:** 10.3390/pharmaceutics16040473

**Published:** 2024-03-28

**Authors:** Nkanyiso L. Ndlovu, Wendy B. Mdlalose, Bulelwa Ntsendwana, Thomas Moyo

**Affiliations:** 1 Discipline of Physics, University of KwaZulu-Natal, Private Bag X54001, Durban 4000, South Africa; 2 DSI/Mintek Nanotechnology Innovation Centre, Advanced Materials Division, Mintek, Private Bag X3015, Randburg, Johannesburg 2125, South Africa

**Keywords:** nanomaterials, ferrite materials, polymeric, nanotechnology, cancer diagnosis, cancer treatment

## Abstract

Cancer is a persistent global disease and a threat to the human species, with numerous cases reported every year. Over recent decades, a steady but slowly increasing mortality rate has been observed. While many attempts have been made using conventional methods alone as a theragnostic strategy, they have yielded very little success. Most of the shortcomings of such conventional methods can be attributed to the high demands of industrial growth and ever-increasing environmental pollution. This requires some high-tech biomedical interventions and other solutions. Thus, researchers have been compelled to explore alternative methods. This has brought much attention to nanotechnology applications, specifically magnetic nanomaterials, as the sole or conjugated theragnostic methods. The exponential growth of nanomaterials with overlapping applications in various fields is due to their potential properties, which depend on the type of synthesis route used. Either top-down or bottom-up strategies synthesize various types of NPs. The top-down only branches out to one method, i.e., physical, and the bottom-up has two methods, chemical and biological syntheses. This review highlights some synthesis techniques, the types of nanoparticle properties each technique produces, and their potential use in the biomedical field, more specifically for cancer. Despite the evident drawbacks, the success achieved in furthering nanoparticle applications to more complex cancer stages and locations is unmatched.

## 1. Introduction

Chronic diseases are a global leading cause of high mortality and morbidity rates [1,2,3,4,5]. According to the World Health Organization (WHO), chronic diseases are prevalent in developed and developing nations and threaten livelihoods globally [6,7]. However, rising chronic disease mortality rates have been observed in developing nations due to their transition from low-income to middle-income status [8,9]. The WHO declared that chronic diseases are composed of four primary categories, which include diabetes, cancer, chronic respiratory disorders (including chronic obstructive pulmonary disease and asthma), and cardiovascular diseases such as heart attacks and strokes [6,9]. An estimated 41 million individuals between the ages of 30 and 69 years are reported to have succumbed to cardiovascular diseases and cancer each year [7,10].

The overall mortality rate may have dropped, but cancer-related deaths have continued to have a linear rise over the years. Recently, more than 19.3 million new cases of cancer were identified and reported based on the available data, resulting in almost 10 million fatalities in 2020 alone [6,8]. The necessity and desire for powerful medications to treat various malignancies has been urged by the ongoing global rise in cancer rates, which claim millions of lives each year. This rise in mortality rates further threatens the progress towards the 2030 WHO agenda for sustainable development goals, which include reducing premature chronic deaths by at least one-third [5,8,10]. Nanotechnology interventions have shown promising results in declining mortality rates for specific types of cancers, with developed countries having carried out most of the clinical trials [6,11]. However, its limitations are less when compared to the drawbacks of traditional cancer therapies [6]. Nanotechnology is a multidisciplinary field with great proven potential in energy, environment, and medicinal areas [12,13,14]. Many nanomaterials have been and are being explored. There is an exponential growth observed in the knowledge of nanomaterials showing multi-functional suitable properties and reduced limitations. This review reports on various uses of nanomaterials in medical applications. It specifically reviews the literature on iron oxide-based nanoparticles and their limitations. The synthesis methods of iron oxide nanoparticles are also reviewed.

### Applications of Nanotechnology for Cancer

The contrast between conventional and modern cancer therapy options allows for a comparison of their applications and their effectiveness. The most administered traditional therapy methods are surgery, chemotherapy (chemo), and radiotherapy. Surgery therapy involves the surgical resection of the infected cancerous cells (tumour) site, relieving the diagnosed individual from effects posed by the tumour and potentially hindering the cancerous cells from further metastasizing throughout the body. This comes at a great loss of organ functions, limbs, and potentially unaffected healthy cells around the tumour. Another commonly used treatment is chemotherapy, which involves using drugs to enhance the immune system in fighting cancerous cells. The type of drug used depends on the diagnosed cancer type, and the mechanism of action may differ with each type of drug. Despite the effectiveness of some of the chemotherapy drugs currently on the market, numerous side effects are still reported. Common side effects include excessive fatigue, hair loss, nerve damage, migraines, diarrhoea, and mucositis [11,15]. Radiotherapy is more of a mechanical therapy through exposing the individual to a low or high dose of beam radiation, which shatters fast-growing cancer cells [16]. Hence, scientists continue to seek convenient ways to fight cancer. Nanotechnologies and nanomaterials have proven to be a great deal regarding the resuscitation of the health system, improving the quality and durability of human lives. The literature reports that nanomaterials possess physical, chemical, and magnetic properties that prolong drug circulation, resistance, tolerability, and improvement, potentially in a manner better than that of traditional materials [17,18,19]. Various characterization techniques are employed in the evaluation of phase, morphological, structural, particle size and surface area, optical, and physicochemical properties of the nanoparticles (NPs).

**Figure 1:**
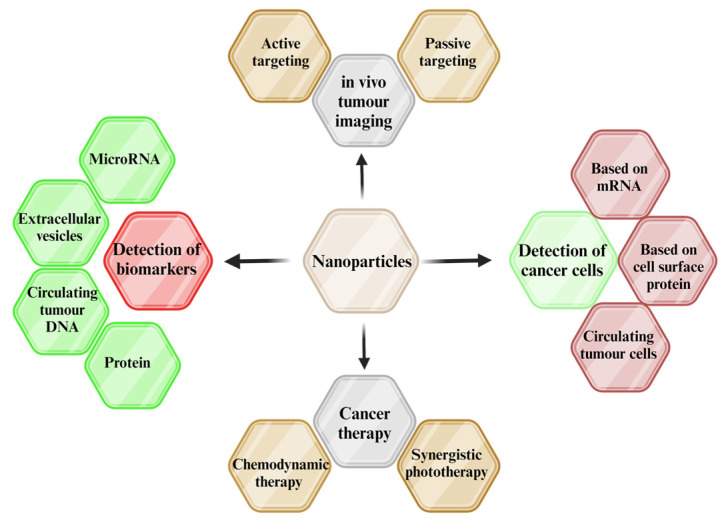
Schematic representation of nanoparticle applications in cancer theragnostic strategies (created with BioRender.com (accessed on 21 February 2024)).

There are selective nanomaterials that possess properties fitting the needs of various fields. Crystallite size and phase confirmation using X-ray diffraction (XRD) are commonly the first points of reference before morphological evaluation. Morphology is of great interest as it influences some of the other properties to be evaluated. Scanning electron microscopy (SEM) and transmission electron microscopy (TEM) are most commonly employed in morphological studies. These techniques provide an understanding of NP shapes, elemental composition (purity), agglomeration (nucleation), and roughness; the homogeneity dispersion of NPs is of importance in applications that require diversely shaped NPs in the form of spheres, nanorods, nanowires, etc. Physical properties such as melting point, transmission, and adsorption of radiation gases by NPs are of importance in applications such as sensors, energy storage, and biomedical applications because these may involve various NP interactions with electromagnetic radiation, electrical, and electronic mechanisms. Physicochemical properties include electronic, optical, magnetic, mechanical, and thermal properties. These are essential to understand because they are the basis of overlapped applications studies for domestic uses in agriculture and industrial purposes. Figure 1 shows various applications of NPs for cancer treatment, diagnosis, and therapy. Additionally, NPs are used as drug carriers, for release strategy and delivery, biomarker mapping, molecular imaging, and the detection of cancer in diagnosis and therapy. Nanotechnology, over recent decades, has opened a wide spectrum of possibilities for NP applications across all fields, particularly in biomedicine.

Significant attention has been focused on the use of nanotechnology for cancer diagnosis and therapy, as alternatives to conventional techniques. NP use provides convenience, high efficacy, cost-effectiveness, and long-lasting and less time-consuming applications [19,20,21]. The nanobiotechnological use of nanoparticles to customize nanomedicines to deal with the malignancy of cancerous cells has shown tremendous potential across the field, having now revolutionized the diagnosis and therapy of cancer treatment [22]. This has received much attention by differentiating the types of NPs suitable for in vivo tumour imaging (passive or active targeting), detection of biomarkers (proteins, extracellular vesicles, circulating tumour DNA, and microRNA), and the detection of cell carriers (circulating tumour cells, based on cell surface proteins and on mRNA) [23,24,25]. These three broad applications form part of the now extensively studied branches of mastering and finding NPs that are part of precision applications in biomedicine. Additionally, various clusters of NPs and the majority of the diagnosis and applications accelerated by nanotechnology require specific NPs to form part of the successful advancement of current and future techniques in curbing various chronic diseases that affect humans. Table 1 shows some NP-based pharmaceutical drugs that have been employed and proven a potential in clinical applications. Advantages of NPs include biocompatibility, longer duration of circulation, increased surface area, and greater adsorption capacity, compared to macroscopic or microscopic materials. Some of the shortcomings experienced with NPs are also presented in Table 1.

## 2. Synthesis Methods of NPs

**Figure 2:**
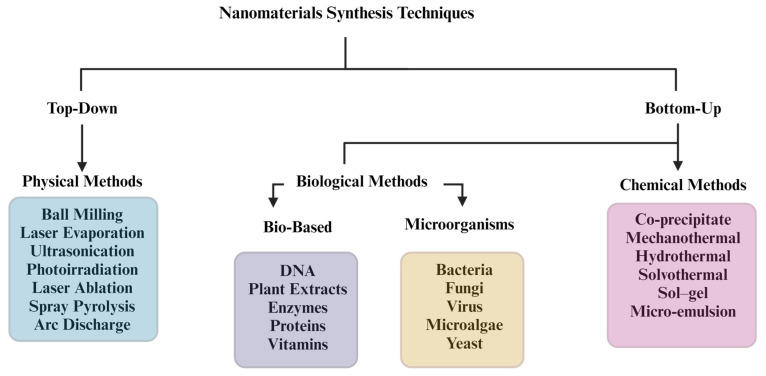
Nanomaterial synthesis techniques and strategies (created with BioRender.com (accessed on 21 February 2024)).

In the past, a great deal of research has been focused on iron oxide-based nanomaterials that include different metals and exhibit a variety of physical, chemical, and magnetic properties [56,57,58,59,60,61,62]. In the literature, it is reported that the synthesis of NPs is categorized into two main groups that differ in synthesis principles, but later produce similar NPs. These are top-to-bottom (top-down) or bottom-to-top (bottom-up) strategies [56,57,63,64,65]. Each strategy has at least one category (biological, chemical, and physical) that is subdivided into a number of expensive, non-expensive, difficult, and easy synthesis techniques. Figure 2 represents various synthesis strategies and techniques utilized for producing nanoparticles. Due to these numerous approaches, we have nanomaterials that consist of numerous property variations [63,64,65,66]. The techniques have flexibility, which enables us to control the factors arising as limitations in the complex functionality necessary for various applications. The choice of techniques plays a vital role in the synthesis of the intended materials [67]. Nanomaterials’ shape, size, structure, crystallinity, yield, magnetization, surface-to-volume ratio, and toxicity properties largely depend on the synthesis techniques. The most common methods used in achieving these properties are given in Figure 2. These methods have proven to be prominent, convenient, simpler, and reliable in the synthesis of the desired NPs [65,67,68]. The synthesis strategies have their advantages, and disadvantages, with the top-down approach being more convenient, producing a high mass yield of NPs, but commonly having surface imperfections. In contrast, bottom-up synthesis is feasible for the creation of nanocluster NPs, which come at a low yield and are quite expensive [69].

The most important properties of nanoparticle preparations comprise particle size and charge, core and surface properties, shape, flexibility, multivalency, and controlled synthesis, as these properties determine the nanoparticle’s in vivo distribution, targeting ability, and toxicity [70,71]. In therapy, these properties have been reported to strongly impact drug loading capacity and release, as well as the stability of the NPs [72]. The impact of nanoparticle size on its in vivo behaviour is one of the most highly investigated aspects of nanoparticle pharmacokinetics and biodistribution [73]. It has been accepted that 10–100 nm is the ideal size for drug delivery systems [74]. Furthermore, the small particle size increases accumulation and enhances the penetration into tissue. Thus, nanoparticle size and surface composition are important determinants of achieving effective target site accumulation.

Furthermore, a controlled synthesis will ideally create nanoparticles with identical size, shape, and charge properties. Hence, in the case of functionalization, an equal number of functional groups bound to the particle’s surface should be sought, if uniform distribution is required. Several alteration occasions and surface modifications exist to influence the in vivo behaviour and amend nanoparticle biodistribution. One of them is to coat the surface area of the nanoparticles with a biocompatible polymer. Polyethylene glycol (PEG) is a frequently used coating material for modifying the surface of nanoparticles. PEG molecules form a protective hydrophilic layer, helping to eliminate recognition by the immune system, thereby reducing the uptake of PEG-coated nanoparticles by macrophages of the magnetic nanoparticles. This process enhances their circulation half-life and subsequent accumulation in target tissues. Other well-known and widely used coating materials are chitosan and dextran [75,76]. Furthermore, chitosan-coated nanoparticles are also used in drug delivery systems [77].

Another effect of nanoparticles is their toxicity and cytotoxicity in healthy cells [78]. Unavoidably, nanoparticles are potentially toxic to the human body. Therefore, nanoparticles must undergo severe screening and testing before being applied to clinical practice [79]. The toxicity of the nanoparticles is an important consideration for the choice of nanocarriers. Hence, the research on this topic continues to rise. Several studies have revealed that the toxicity of nanoparticles is related to size, shape, and concentration [78,80,81]. However, surface coating has been one of the most popular surface modification strategies to reduce the possible toxicity of nanoparticles, which is reversible using non-covalent modification [67]. Surface coating can alter the dispersion state of nanoparticles, which significantly determines their bioavailability and potential toxicological effects [82]. Hence, it is remarkable that the properties of the nanoparticles suitable for different applications depend mainly on the synthesis method. The different synthesis methods are discussed below with a schematic representation and the typical obtained transmission electron microscopy (TEM) and scanning electron microscopy (SEM) images of the resulting NPs.

### 2.1. Biological Method

**Figure 3:**
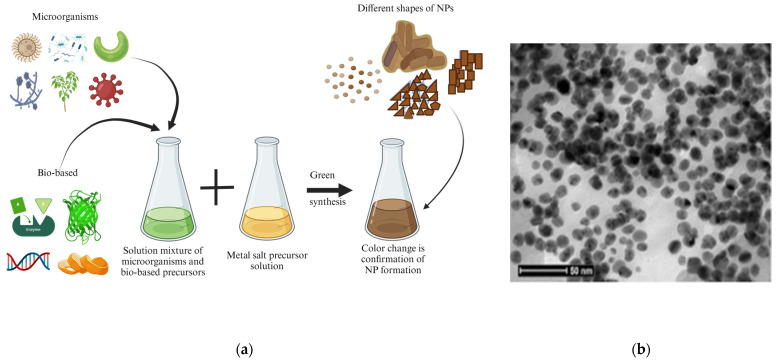
(**a**) Typical schematic for biological NP synthesis from natural precursors (created with BioRender.com (accessed on 21 February 2024)). (**b**) Silver NPs TEM image synthesised using green methods [84].

The biological synthesis method is a “new” alternative possibility to obtain NPs in an economical and environmentally friendly way. Additionally, it is an easy strategy to follow. It is a branch of bottom-up synthesis which only involves green synthesis approaches; the typical synthesis methods and steps involved are represented in Figure 3 for acquiring various types of nanoparticles [83].

There have been comparatively fewer reports in the literature on biological agent-assisted synthesis because, although it is a more environmentally friendly method, these NPs may be less stable and non-uniform, with less homogeneity and more agglomeration [85]. Recently, Hamdy et al. [86] reported, in a review, on numerous iron oxide-based NPs with properties that are potential for biomedical applications. Patil et al. [83] also reported a synthesis of numerous NPs and their various applications. Their anticancer and biosensing applications are currently critical in the alleviation of a worldwide chronic disease [83].

### 2.2. Chemical Methods

Chemical methods, also known as “wet chemistry”, are the leading, most unique and reliable synthesis routes for new nanomaterials with intriguing properties. These techniques allow for us to monitor or control one or more synthesis factor, such as concentration (of reactants), pH, heat, capping agents, base, and atmospheric conditions (inert or oxygen-free system), that can affect the desired properties of NPs [87]. However, just like every other procedure, there are routes that remain preferable over others, which are discussed below.

#### 2.2.1. Co-Precipitate Method

**Figure 4:**
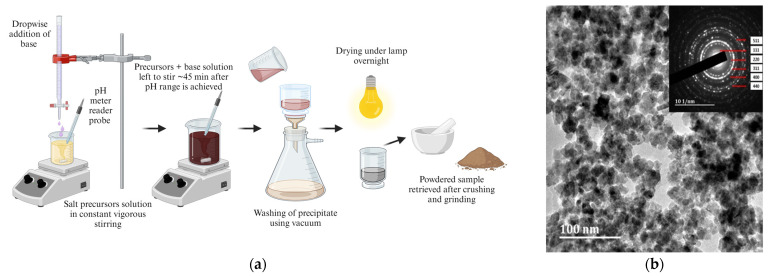
(**a**) Schematic representation for co-precipitate synthesis method of NPs (created with BioRender.com (accessed on 21 February 2024)). (**b**) HR-TEM image of cobalt ferrite nanoparticles.

Co-precipitation is one of the easiest and most convenient, commonly used synthesis approaches due to its flexibility of reaction conditions, such as temperature, atmosphere, yield, solvent variations, and pH control, to achieve improved variations of physical, chemical, and magnetic properties [67]. Nevertheless, due to its stages occurring simultaneously, it makes it very difficult to prevent agglomeration from its nucleation of NPs forming, which heavily affects the shape, size, and magnetic properties [69,87]. The steps involved in the co-precipitate synthesis method are represented in Figure 4.

However, unlike the top-down strategy, wet chemistry reactions, before exposure to varying temperatures, have proven to be promising in controlling synthesis factors. Nanomaterials that have properties close to the desired characteristics suitable for biomedical, biosensing, targeted drug delivery, and magnetic hyperthermia treatment for cancerous tumours have been reported, among various applications [88,89]. The Fe_3_O_4_ NPs with high stability and a size range of 16–19 nm, determined from the XRD patterns, are reported in the literature as better than those synthesized using a top-down strategy. Additionally, Fe_3_O_4_ NPs were synthesized using the co-precipitation method for evaluation of their effectiveness in wastewater treatment removal of turbidity, achieved using pH control and the size of NPs [90]. Andhare et al. [91] reported on the single-phase cubic spinel structure of zinc–cobalt ferrite (Co_(1−*x*)_Zn_(*x*)_Fe_2_O_4_) NPs on the effect of zinc (Zn) doping on morphology, size, and chemical and magnetic property variations as Zn concentration is increased. The authors observed the physical and magnetic properties increased and decreased, respectively [91].

#### 2.2.2. Sol–Gel Method

**Figure 5:**
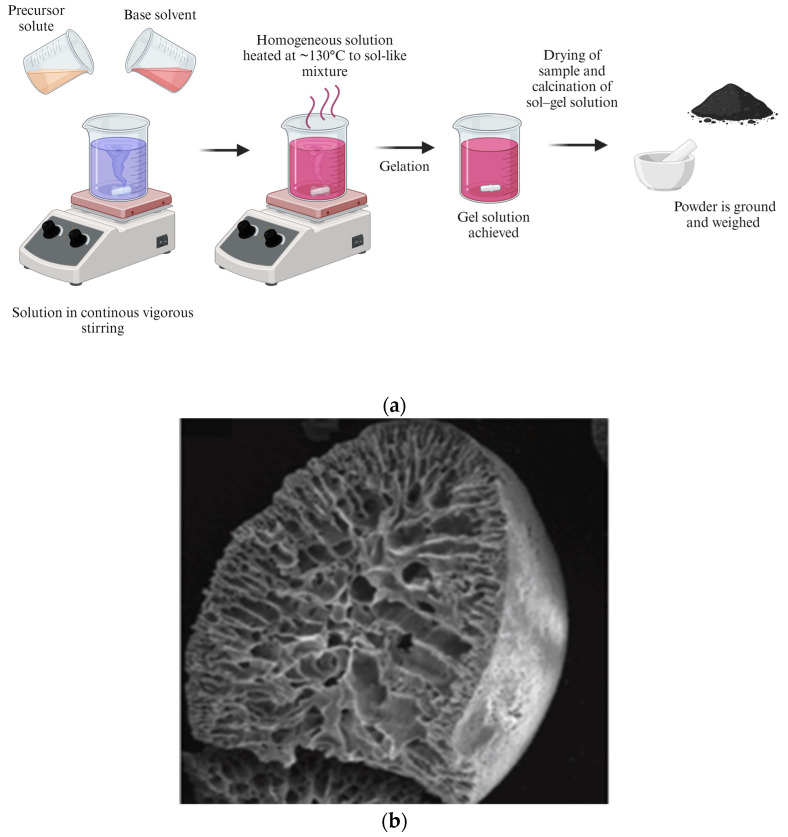
(**a**) Sol–gel schematic synthesis representation (created with BioRender.com (accessed on 21 February 2024)). (**b**) Typical TEM results of NPs synthesized via this method [93].

The sol–gel synthesis method is a way of preparing nanostructured metal oxides and mixed metal oxide nanocomposites [92]. The name “sol–gel” comes from the process of forming nanomaterials from solution (sol) into which colloidal particles are dispersed. A “gel”, which is a semi-solid or continuous solid network forms by a process of gelation, as shown in Figure 5.

The method comprises a few steps in the process of synthesizing a metal oxide nanostructure, which are the hydrolysis, condensation, ageing, drying, and calcination of the precipitate [94,95]. One of the drawbacks of this synthesis method is that the steps involved in the synthesis can all take place at the same time, making it difficult to synthesize the nanostructured metal oxides in a controlled manner. It is sometimes advantageous to coat magnetic nanomaterial with a material that is non-toxic and that can support the required amount of the magnetic nanomaterial, of which the silica as a host protects and stabilizes the magnetic nanomaterial and, in addition, it also prevents any contamination [92,94,95].

#### 2.2.3. Thermal Methods

**Figure 6:**
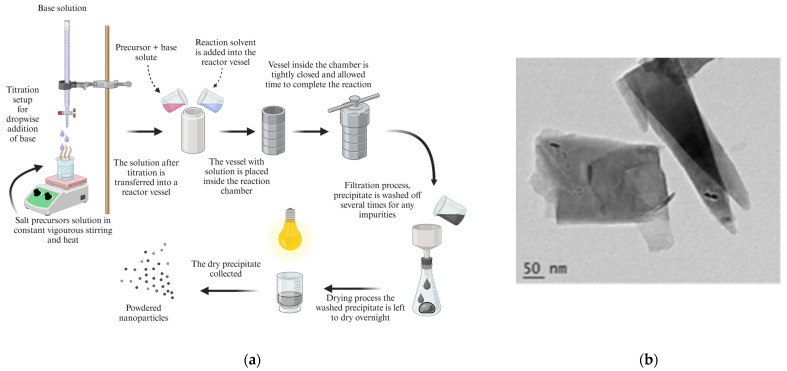
(**a**) Typical thermal synthesis method for NPs (created with BioRender.com (accessed on 21 February 2024)). (**b**) TEM image of zinc oxide nanorods prepared using the hydrothermal method [98].

Thermal methods are considered as important approaches to inorganic synthesis because of their low-cost, high-speed, and environmentally friendly properties, in addition to their growing potential as current and future advanced synthesis methods for the modifications of NPs for various applications [69,96]. Hydrothermal, solvothermal, and glycol-thermal synthesis approaches are major chemical processes in aqueous or nonaqueous solutions that take place at comparatively high temperatures well above the boiling point of water. The synthesis via hydrothermal and solvothermal steps shown in Figure 6 are similar and differ only by the solvent used for the precursor solution mixture. These techniques have been widely used in the syntheses of advanced materials because they have the advantage of being straight forward, easy, effective, and inexpensive [67,97]. NPs possess higher melting point temperatures in comparison to their precursors, which enables the use of higher temperatures to eliminate unnecessary compounds such as ethanol and water from the prepared NPs through thermal methods, without changing the chemical structure of the nanomaterials [96,97].

##### Hydrothermal Method

The hydrothermal method is a technique that uses deionized water (hydro) as a solvent for precursor mixing and heat (thermal) treatment to an airtight pressure vessel under pre-set or controlled pressure at 100 °C temperature for a specific time lap in the formation of complexes, as shown in Figure 6. Synthesis can also be performed at atmospheric pressure in a round-bottomed flask using a heating mantle and a tap-water-cooled condenser. This technique is common in the synthesis of important solid preparations such as luminescence phosphors, superionic conductors, and microporous crystals. It is also a route to unique condensed materials, including thin films, nanometre particles, and gels [99]. Gómez et al. [100,101] reported magnetite synthesis with well-defined morphology, and morphology control was achieved by varying temperature using the hydrothermal synthesis technique [100,101] A comparative study was conducted by Jesus et al. [99] between co-precipitation and hydrothermal methods, with the addition of a capping/chelating agent to form a more stable metal ion complex reported with a very small size distribution (3–5 nm) and well-defined morphology, and the hydrothermal method is, comparatively, a better synthesis route than co-precipitation, despite both being wet chemistry techniques.

##### Solvothermal Method

The solvothermal technique setup, as shown in Figure 6, differs by the nonaqueous solvent used in its reaction for the crystallization process. Apart from that, thermal techniques are similar as they take place under similar conditions, and variations are attributed to the optimization of the growth parameters. The solvothermal method of synthesis occurs in a sealed vessel at higher temperature than 100 °C. Yan Hulo et al. [102] reported on the synthesis and the applications of various NPs and their comparative growth changes to further elucidate optimization parameters for NP properties [102]. Sangapath et al. [103] reported promising results for the application of nanotechnology in biomedicine due to their ability to facilitate surface functionalization with bio-compatible polymers.

##### Glycol-Thermal Reaction

**Figure 7:**
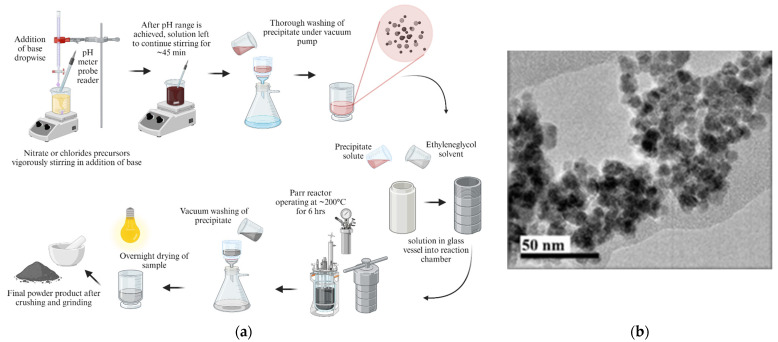
(**a**) Glycol-thermal schematic synthesis method (created with BioRender.com (accessed on 21 February 2024)). (**b**) Typical TEM image of glycol-thermal reaction prepared NPs [106].

The schematic of the glycol-thermal technique is shown in Figure 7. This is a wet chemistry method similar to co-precipitation. This method involves a mixture of stoichiometric salts dissolved in de-ionized water as solvent, to form a solution of precursors. This is followed by a thorough monitoring of the pH as base is added (dropwise) to form a precipitate, under continuous vigorous stirring of the solution until a complex of desired pH range is achieved. Then, the solution is washed several times to remove chloride or nitrate ions. The precipitate is reacted in ethylene glycol solvent in a high-pressure reactor at 200 °C for at least 4 hours under continuous vigorous stirring [104,105]. After synthesis, the sample is washed, dried, and is ready for characterization.

### 2.3. Physical Methods

The physical method is the only one of the various synthesis techniques (see Figure 2) with a top-down strategy. This involves having bulk materials as precursors to produce nanomaterials using larger and externally controlled devices to break down the bulk materials [96]. As a result of the immense focus on the fabrication and application of NPs, synthesis via mechanical ball milling stands out to be convenient and cost-effective in obtaining the desired NPs.

#### Ball Milling

**Figure 8:**
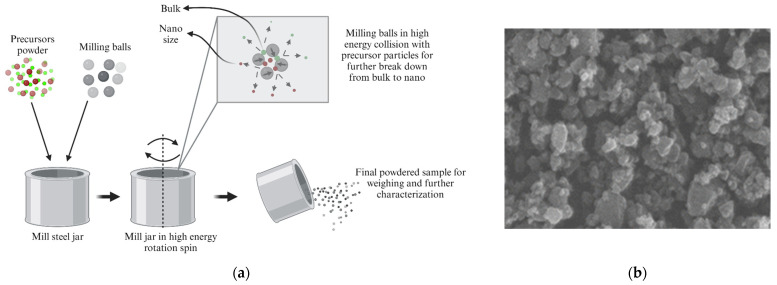
(**a**) Ball milling method for synthesis of nanomaterials (created with BioRender.com (accessed on 21 February 2024)). (**b**) SEM image of copper oxide nanocomposite synthesised using ball milling [112].

The high-energy ball milling or mechanochemical milling (ball milling) method is one of the easiest, most promising, and most time-consuming strategies, operating on the principles of the exploitation of high energy to reduce the bulk materials through impact and reduction [107]. The composition of precursors for the desired complex is mixed together in a calculated mass-to-ball ratio for an effective and uniform dispersion of the powdered NPs. When the balls gain kinetic energy inside the vessel, they randomly move and collide with the sides of the vessel or with another ball, creating an impact, which then breaks down the bulk nanomaterials and results in size reduction; the mechanism is shown in Figure 8 [108,109]. This is a commonly utilized technique in physical methods. Additionally, it is also a joint technique for the coating process of the prepared NPs for their functionalization.

Ball milling is highly recommended for its cost-effective usage and successful synthesis of NPs. It does not require precipitation or washing procedures. Several reports are available in the literature that evaluate the effects of milling time, mass-to-ball ratio, and surface functionalization. Progress towards single phase formation can be monitored at regular time intervals using XRD. This is important because milling for a less or longer time could easily result in incorrect or destroyed desired phase formation [103,109,110,111]. Other disadvantages of the technique include contamination of the samples from the surfaces of grinding balls and jar, as well as a slightly higher range of particle size distribution, due to limited ball sizes.

## 3. Suitable NPs

Cancer nanotechnology aims to define the interaction of nanoscale applications with cellular and molecular components compatible with cancer detection and treatment. The ability to retain the chemical properties of their bulk materials is beneficial in selecting NPs for various applications based on the following three classes of materials viz: carbon-based, organic, and inorganic types of nanomaterials. Each class has various nanoparticles showing promising potential to develop unique therapeutic qualities due to their superparamagnetic nature. This enables the ability of NPs to infiltrate tumours deeply and with great specificity by using magnetic fields [113,114,115].

### 3.1. Carbon-Based NPs

The thermal, mechanical, electrical, optical, and structural variety of chemical and physical properties of carbon-based nanomaterials (CBNs), comprising carbon nanotubes (CNTs), graphene oxide (GO), and graphene quantum dots (GQDs), have been intensively researched for various cancer applications due to their intrinsic qualities [116].

The synthesis of CNTs may yield single- or multi-walled, layered nanoparticles of tubes with flexible helical structural shapes, functionalized with biocompatible polymers for potential cancer diagnosis as biosensors, therapeutics agents in targeted diseases (AIDS, viral infections, and tumours), and in vitro for pre-clinical models [116,117]. A detailed review of the CNTs for biomedical applications has been reported by Maiti et al. [116,117,118] and Kapil Patel et al. [116,117], who have highlighted their theragnostic potential, due to their unique and diverse properties.

Fullerenes, also known as “buckyballs”, are paramount carbon-based nanocomposites, owing to their stability as crystallites in the NPs size range of 25–500 nm. They are promising in applications as they can deliver drugs or small therapeutic molecules to cancer cells. Functionalized fullerenes possess characteristics that support anticancer therapy, apart from tumour inhibition, and help combat systemic toxicity and drug resistance, which are commonly encountered in the conventional chemotherapeutic approach. Hosnedlova et al. [116,119,120] have reported a critical review of CNTs’ various forms, shapes, structures, functionalizations, and their applications in cancer treatment [116,119,120].

### 3.2. Organic Types

Organic nanomaterials have been widely investigated in the literature. They include various types of micelles, liposomes, ferritins, polymersomes, nanogels, exosomes, polymeric nanoparticles, and dendrimers. They are known to be nontoxic, biodegradable, and eco-friendly. Hollow-cored liposomes and micelles are light-, heat-, and electromagnetic radiation-sensitive [121,122]. Organic NPs are ideal as drug delivery agents due to the three-dimensional internal holes capable of holding more substitute molecules [122]. Additionally, they are highly recommended for optimal applications in the biomedical, energy, electronics, sensing, imaging, and nanocomposite systems. This is due to their high biocompatibility, stability, load capacity, delivery efficiency, and surface morphology. The control of NP dispersions and interactions in materials over broad areas remains a challenge. A full understanding of simple, efficient, and controlled techniques for mass production is promising [123]. The evaluation assay on the applications of nanomaterials is crucial for a longer life. Gulumian et al. [124] reported the current risk evaluation of nanomaterials and this research revealed the benefits of organic-type materials to be suitable for various applications.

### 3.3. Inorganic Types

In comparison to organic materials, inorganic nanoparticles are considered to be moderately toxic, hydrophilic, biocompatible, and very stable. Inorganic NPs can be in the form of elemental metals, metal oxides, and metal salts. In terms of advanced biomedical materials, superparamagnetic iron oxide nanoparticles (SPIONs) have a distinct superparamagnetic nature. When SPIONs are subjected to an external magnetic field, a significant magnetization can be generated, which then vanishes when the external magnetic field is removed. Because of a large magnetization, there is a greater signal change per unit of particles. This leads to lower amounts of particles required to generate excellent signal feedback used for imaging, as well as for drug and gene delivery strategies. Numerous articles have focused on current research studies, showing the advantages of inorganic nanomaterials for in vivo treatments, controllable physicochemical properties, and easy surface modification for multi-functionalization [125,126,127].

#### 3.3.1. Metal Iron Oxides

The metal iron oxides in nanotechnology have been of great interest for studies of materials with small or ultrafine sizes in the range of 1–100 nm. Interest persists due to the multidisciplinary applications of these minerals in medical imaging, drug targeting, catalysts, gas sensors, optical devices, refrigeration, biosensors, magnetic recording media, and other biological applications. Metal iron oxide NPs, in comparison to their counterpart bulk materials, have enhanced molecular interactions. Particles in this nanoscale range exhibit significant size-related properties such as easy adsorption, improved absorption, and easy penetration. Metal iron oxides are well known for their natural occurrence in nature. Simple synthesis routes can produce high yields of precipitates, distinguished by their reddish brown/black colour. There are three main types of iron oxides, these being magnetite (Fe_3_O_4_), ferric oxide (Fe_2_O_3_), and ferrous oxide (FeO). Due to their size-related properties, numerous publications have been reported on high-quality iron oxide NPs with controlled morphology; some significant results have been observed in wastewater treatment in the removal of organic pollutants, Mokhosi et al. [67,87] and Sajid et al. [67,87]. Iron oxide-based NPs have been tested in clinical trials for various cancer treatments.

#### 3.3.2. Ferrite Materials

Ferrite magnetic nanoparticles (FMNPs) are spinel ferrite microcrystallites with a cubic crystal structure. Depending on the cationic distribution in the tetrahedral and octahedral sites, the ferrites are distinguished into three types of spinels (normal, inverse, and mixed) structures. These compounds are identified by a well-known chemical formula, *M*Fe_2_O_4_ (*M* = Cu, Co, Mn, Zn, and Ni), where *M* is a divalent metal ion and forms the basis of many nanotechnology applications [128]. The size, crystallinity, compositions, and surface-to-volume ratio are important properties of ferrites for functional magnetic and physicochemical applications. However, although ferrites are easy to synthesize, the tuning of their properties depends on the synthesis method used and parameters such as precursors, surfactants, reaction time, temperature, and pressure. These magnetic metal-oxide materials have been known for several decades as ferrimagnetic ceramics used in electric and optoelectrical devices due to their high electrical resistance and low eddy currents losses [128].

#### 3.3.3. Rare Earth Metal Iron Oxides

The rare earth (RE) elements have atomic numbers ranging from 57 up to 71 for La to Lu on the periodic table [129]. Rare earth elements are divided into the following two groups: lighter (lanthanum to gadolinium) and heavy (terbium to lutetium) metals [129,130]. They all have the same inner shell electronic configurations of [Xe] 6s^2^, but differ only in the occupation of 4f and 5d, between 4f^0^ 5d^1^(La) and 4f^14^ 5d^1^(Lu). The predominant oxidation state is 3, followed by 2, and, additionally, 1 or 4 oxidation states for a few elements. They offers a wide range of chemical, physical, and magnetic properties associated with RE elements, which account for a diverse range of application devices such as smartphones, digital cameras, computer hard disks, and fluorescent and light-emitting diodes. RE cations are beneficial in metal–metal compounds. They can be used as dopants or to substitute other cations in order to improve properties. The 4f electrons have a significant contribution to magnetic moments and hence contribute to unique changes in magnetic properties. In medical applications, the literature focuses on combinations of promising anticancer compounds that will produce minimal or no side effects [12,129,130,131]. Table 2 summaries the various applications of carbon-based, organic, and inorganic types of NPs and their limitations.

## 4. Functionalization of NPs

Nanotechnology research has paved the way for the functionalization of NPs as an answer to limitations posed by NPs in various applications. Functionalization is a process of NP surface modifications with chemical conjugates or molecules onto the surfaces of NPs to enhance or improve their uptake efficiency and biocompatibility for theragnostic purposes [154]. Iron oxide-based NPs are prone to diminished stability in colloidal solutions or biological fluids. Surface functionalization is, therefore, key in solving this problem. These NPs are hydrophobic and positively charged with high surface energy (because of their high surface-to-volume ratio), resulting in agglomerates [155]. Various functional polymers and polysaccharide species are used as coating agents. These polymers include polyethylene glycol (PEG), polyvinyl alcohol (PVA), polyacrylic acid (PAA), polyethyleneimine (PEI), and chitosan [155]. This is carried out without altering the physical and magnetic properties of the NPs, but improves their resistance to erosion, shape, and the controlled leakage of magnetic core materials after application, and offers colloidal stability to the NPs [155]. Asida et al. [156,157] and Gambhir et al. [156,157] have reported on the synthesis and latest functionalization states of the biopolymer-mediated magnetic iron oxide-based NPs with enhanced properties for biomedical (cancer) therapy applications.

## 5. Diagnostic Methods

Imaging has been a strong tool in the diagnosis of cancer. Magnetic resonance imaging (MRI) and computer tomography (CAT) scans have made significant contributions to diagnosis advances. However, nanotechnology now provides an alternative tool for in vitro and in vivo diagnostics techniques that is sensitive and extremely accurate, way ahead of the capabilities of traditional methods. Nanotechnology is understood to provide cellular and sub-cellular diagnostics [5,6,158,159]. It allows health professionals to detect, reduce, and possibly eliminate malignancies as soon as possible.

### 5.1. Tumour Circulation

**Figure 9:**
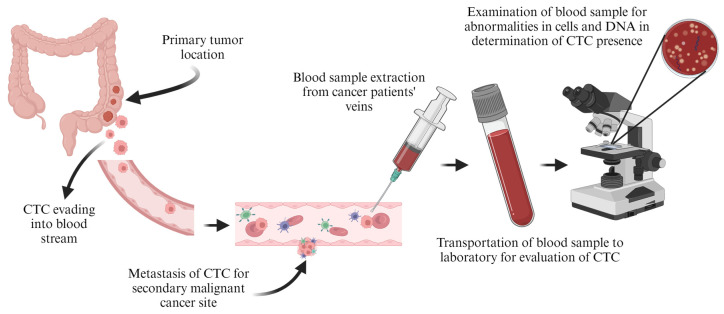
Representation of CTC from primary tumour to secondary metastasis (created with BioRender.com (accessed on 21 February 2024)).

In 1869, Ashworth [158] observed the circulation of tumour cells (CTCs) from cancer-diagnosed patients’ bloodstream. In this study, single or clusters of CTCs that split away from the primary tumour were observed to spread and metastasize in the entire body. Figure 9 shows a typical tumour circulation from primary to secondary metastasis. The CTC detection forms part of the in vitro techniques, through which extracted blood samples are evaluated for any abnormalities.

The detection of CTCs is easily conducted through physical isolation by the size of epithelial tumour (ISET) cells, which have a bigger diameter than that of white blood cells. The effectiveness of this technique is minimal due to the ability of cancer cells to remain dormant for years, independent of the primary tumour, so chances of blood extraction and successfully detecting the CTCs are slim, though an early detection would mean early stages of cancer and better chances of being diagnosed and treated early [158].

### 5.2. Imaging

Advancements in imaging tools, which produce images of the body, are essential components to the early diagnosis of various malignancies. However, the importance of imaging does not only lie in its detection (screening) capabilities, but also crosses over to depict the stages of cancer, the specific location of the advanced tumour cells, determining if treatment is effective, and monitoring if there is any cancer recurrence. With this information, improved proper diagnosis and appropriate treatment methods at early stages can prevent further metastasis and ensure effective treatment [160,161,162].

An X-ray scan is one of the familiar imaging techniques generally employed to look for a fracture and absorption rates of tissues in radiographs and mammograms, for early upper body (chest/breast) cancer detection and diagnosis. The X-ray exam is performed to observe any obvious primary abnormalities or CTCs spread in the upper areas of the body. While CAT scans operate on the same basis as X-rays, they differ in the information they collect. Both confirm the presence and location of malignancies, but the technique that provides precise information about the depth of location is the CAT scan. This is advantageous in situations where targeted or surgical treatment is to be employed [161].

Ultrasound, positron emission tomography (PET), single photon emission computed tomography (SPECT), and MRI scans all operate on the basis of transmitted waves to construct images. MRI operates with the use of external magnetic fields and radio waves [162]. PET and SPECT use radioactive tracers to create relevant images.

## 6. Cancer Treatment Strategies

The proper and early detection of cancer promotes early diagnosis and implementation of treatment strategies for cancerous cells. Conventional cancer treatments require precision and accuracy in addressing cancer-infected sites in their developing or primary stages to alleviate and achieve high efficacy and efficiency. This has resulted in more studies being focused on improving and new smart treatment strategies. Advancements in the detection of cancer has paved the way for modern treatment techniques that are dependent or suitable to specific types of tumours and how advanced the cancer is. Although the transition from conventional to modern is not complete, chemo, radiation, surgery, and other modern techniques are used in combination with modern techniques [163]. Some NPs that have been studied and/or gone to clinical trials for evaluations in different cancers are listed in Table 3. The strategies used in the listed literature are discussed below.

### 6.1. Biomarkers Testing

**Figure 10:**
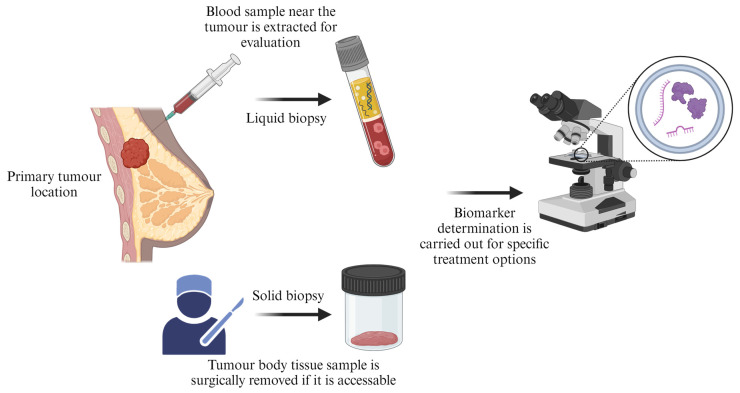
Biomarker for cancer evaluation from a cancer-diagnosed individual (created with BioRender.com (accessed on 21 February 2024)).

Biomarker testing is a measure or indicator for the risk, occurrence, or outcomes of cancer in patients. The application is used to check for tumour biomarker characteristics (genes, proteins, and other substances) that can provide distinguishing information about cancer. Similar to DNA, cancer has unique patterns of biomarkers for each individual diagnosed. The evaluation of cancer presence using biomarkers is represented in Figure 10. Understanding the primary information about tumour “biomarkers” assists in the early detection of certain characteristics present or absent for specific cancerous cells, and an informed decision can be made on a suitable course of treatment.

There are various biomarkers for cancer testing. They vary according to the type of test performed. These include single, multigene, whole-exome, and whole-genome sequencing [172]. Biomarkers form part of the modern therapy of precision medicine, catering for disease prevention, diagnosis, and treatment. Molecular cancer biomarker potentials and future perspective in cancer precision oncology have been reported [173].

### 6.2. Hormone Therapy

Hormone therapy is a simple straightforward technique used to slow or stop metastasizing cancer cells by fast tracking their growth. It is distinguished into the following two groups: one that blocks the body’s ability to produce hormones and the other that interferes with hormone behaviour in the body. In addition, it is an alternative for men diagnosed with prostate cancer who cannot undergo surgery or radiation therapy to ease or prevent symptoms [174]. Deli et al. [175] reported on hormone replacement therapy (HRT) in cancer survivors. The informed decision-making involved in HRT applications considers several factors, such as the general oncological characteristics of the malignant disease, planned hormone substitution therapy specifications, and relevant endocrine characteristics of the tumour. Misconceptions between HRT and menopause hormone therapy remains unclear. Reports on the slight risk increase in the progression of some cancers remains, although HRT remains as an alternative treatment for some cancers and studies are carried out on the advancement of suitable hormones [176].

### 6.3. Photodynamic and Photothermal Therapies

**Figure 11:**
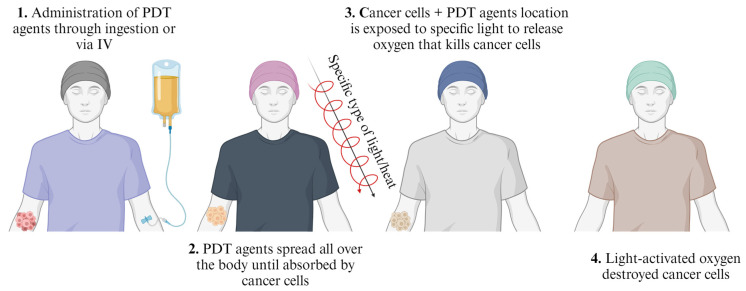
Step wise representation of PDT treatment (created with BioRender.com (accessed on 21 February 2024)).

Light-mediated phototherapy techniques, such as photodynamic therapy (PDT) and photothermal treatment (PTT), have been utilised as viable substitute therapies for several illnesses because of their distinct benefits, which include less invasiveness, enhanced selectivity, and low side effects [177]. The typical PDT treatment steps are represented in Figure 11. PDT and PTT are similar in that they both require oxygen to function. Since reactive oxygen species (ROS) production in PDT requires a functional vasculature and oxygen, PTT is primarily oxygen-independent and is, therefore, well suited for treating hypoxic tumours. While certain medications work better at high temperatures or when ROS are present, the field of assisted PDT and PTT has advanced significantly in recent years, especially in the development of synergistic therapy approaches, such as combining PDT or PTT with chemotherapeutic modalities [178]. PDT uses drugs known as photosensitizers or photosensitizing agents activated by light techniques to release a form of oxygen that destroys tumour cells in treatment. The PDT agents are administered through ingestion or intravenous (IV) methods to spread and be absorbed by tumour cells. Light of a specific wavelength is directed onto the location of the tumour and the agents absorb this light. In return, radical oxygen molecules are released in the photochemical reaction, which destroys the tumour cells. PDT is approved to treat certain cancers, namely advanced cutaneous T-cell lymphoma, Barret oesophagus, oesophageal, basal, and squamous cell skin cancer, non-small cell lung cancer, and actinic keratosis [174]. PDT is a cancer-specific technique and is effectively advantageous if administered early, in cases where other techniques are not suitable or are too advanced. Panaseykin et al. [179] reported the early diagnosis of oral cavity cancer stage 1 for patients with serious side diseases (HIV infection with associated pulmonary hypertension of high degree and cardiac pathology). PDT therapy has been known to successfully destroy cancerous cells, patients are reported to be in full remission and no evidence for progression observed.

### 6.4. Stem Cell Transplant

Stem cells are a therapy technique which has proven promising efficacy in patients who have undergone diagnosis and treatment of cancer. Stem cells serve as first aid cells to restore blood-forming cells that were collaterally damaged in the deterioration of tumour cells, because of high doses of chemo and radiation treatments. These are unspecialized cells. A procedure is performed to specialize these cells for specific functions. These cells are commonly used to treat patients diagnosed with cancers that affect the blood cells, such as leukaemia, lymphoma, multiple myeloma, and myelodysplastic syndromes. In addition, it may be used for neuroblastoma, Ewing’s sarcoma, brain tumours that have come back in children, germ cell tumours, and testicular cancer [174]. Zakrzewski et al. [180] explains the history of stem cell biology and the classifications of available and known stem cell transplants.

### 6.5. Chemodynamic Therapy

In order to identify different proof-of-concept research-based therapy techniques that assist effective suppression of tumour growth, recurrence, and metastasis, a large number of new therapeutic targets and developing therapeutics have been established. Chemodynamic therapy (CDT) is a new type of cancer treatment that involves producing •OH in the tumour location by means of a Fenton or Fenton-like reaction [169]. Iron-containing substances or treatments that release ferrous ions in tumour cells set off the Fenton reaction, which produces •OH (often in an acidic environment) and ultimately results in the death of tumour cells, the basis for the Fenton reaction-based CDT. Because CDT does not require complex therapeutic instruments, treatment expenses are comparatively modest. Many biological researchers have been paying more and more attention to CDT since it was first presented. CDT techniques based on iron have received a lot of attention such as PDT, PTT, sonodynamic therapy, and chemoimmunotherapy [169,181,182].

### 6.6. Targeted Therapy

**Figure 12:**
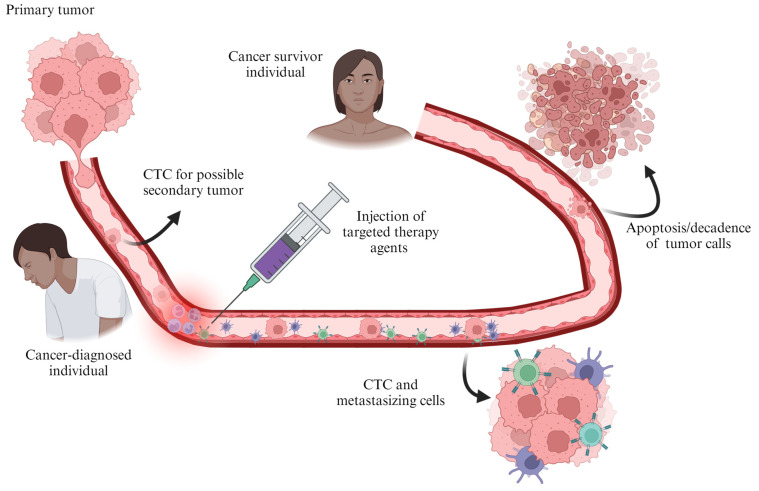
Representation of CTC treatment from a primary tumour from metastasizing (created with BioRender.com (accessed on 21 February 2024)).

Targeted therapy is among the fast-growing advanced cancer treatment techniques employed for various therapies. It works on the principle that it targets the proteins and inhibits proliferation, differentiation, and migration of cells that regulate the growth, division, and metastasis of cancer cells. Targeted therapy forms part of the focused precision medicine’s cornerstone in biomedicine [183]. Researchers are getting better at understanding DNA characteristics that drive the growth and survival of tumour cells, in order to create drugs that specifically counteract in a targeted approach, the proteins and DNA alterations that cause cancer. Figure 12 shows the developments from a primary tumour to a potential secondary tumour that is targeted before potentially metastasizing or a tertiary malignancy. Targeted therapy is based on either small-molecule drugs or monoclonal antibodies, which are similar. However, the monoclonal antibodies are more target-specific and less toxic as a result of binding other targets.

The therapies are administered by ingestion or injection, respectively. Small (NP) drugs promote easy penetration to access foreign cells (tumours). Meanwhile, monoclonal antibodies are lab-produced to highlight precise locations or capture/attachment onto the cancer cells in order to cause self-destruction or to enact the immune system to identify and destroy cancerous cells [174,184]. Various agents have been reported in the literature as a first line of targeted therapies in breast cancer of human epidermal growth factor receptor 2 (HER2). These include trastuzumab, pertuzumab, lapatinib, and trastuzumab emtansine (T-DM1). It has been reported by Oh and Bang [185] that these drugs have been clinically approved as treatment for HER2-positive breast cancer. This has been supported by a detailed review on the HER2-positive metastatic breast cancer [186]. There are significant improvements in prognoses of patients suffering from this disease with new anti-HER2 drugs which are improvements from first, second, and beyond therapies to emerging novel combinations of future treatment techniques [186].

### 6.7. Magnetic Hyperthermia

**Figure 13:**
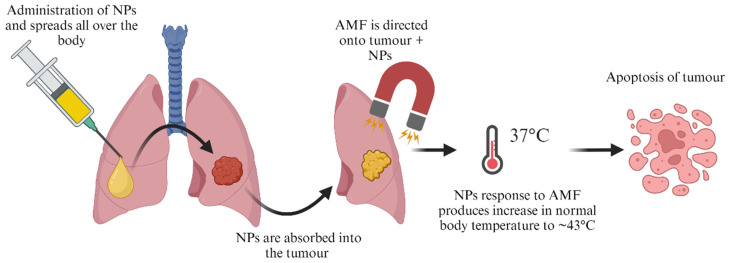
Magnetic hyperthermia treatment of cancer (created with BioRender.com (accessed on 21 February 2024)).

Magnetic hyperthermia (MH) is one of the well-known therapies for cancer, which dates back to the 1950s. It is has been shown to be a significant nanotechnology-based cancer thermal therapy complimentary alternative to existing treatment strategies [187], there is still limited success achieved. Magnetic hyperthermia treatment, as illustrated in Figure 13, is a heat technique in body tissues that uses the incapability of cancer cells to survive temperatures as high as about 43 °C, which destroys tumour cells [188].

MH treatment uses NPs that are small enough, about 10 nm−100 nm in diameter, and have properties that include high heating power and stability and they achieve a superparamagnetic (single domain) state when the temperature is above blocking temperature. The NPs should have high saturation magnetization to allow for easy control of minimizing the magnetic energy generated by an external magnetic field [189,190]. There are numerous synthesis methods to produce coated and doped NPs with promising properties for MH. Unfortunately, very little attention has been devoted to this because of lack of recent reports for application or pre-clinical approved trials. This may be due to the issue of finding precisely suitable synthesis, coated, and doped NPs for MH. However, work is continuing to better understand and explain the biological mechanisms and nanoscale heating from a single-cell level to multiple cells in the body [191,192,193].

## 7. Conclusions

The recent applications of modern nanotechnology as cancer theragnostics have made a significant impact on the projected decline of cancer mortality cases towards the year 2030. Some success in synthesis techniques from the bottom-up strategy has been achieved. NPs with enhanced surface-to-volume ratios, biocompatibility, functionalization, tuning of toxicity levels, and doping or substitution with rare earth metals have been shown to exhibit potential properties for applications. Moreover, the scientists are still working to finding convenient nanomaterials that may be safe to use in applications, such as magnetic hyperthermia, biomarker testing, and targeted drug delivery agents. The advanced conjugated applications have demonstrated a seamless transition from conventional to modern cancer therapies. In addition, it is clear that through a combination of therapeutic methods, many drawbacks could be eliminated. However, the aim is to achieve maximum efficacy and improved efficiency of NPs against cancerous cells with low risks of side effects or damage to healthy cells. As a result, improving the synthesis factors affecting NPs could potentially reduce the shortcomings of NPs and conventional methods. Consequently, more focus still needs to be directed towards achieving precision medicine. This will serve well to find simple, efficient, and easily controllable ways to produce nanomaterials for mass production, as well as to bridge their applications.

## Figures and Tables

**Table 1:** Summary illustrating various NP drugs synthesis, their approaches, applications, and their limitations.

Type of NPs/Drug/Name	Method of Synthesis	Application/s (Advantages)	Limitation/s (Disadvantages)	References
IONP@PMSEA	Thermal decomposition	Potential delivery agents for therapeutics and diagnostics	Reproducibility and scalability	[26,27,28]
Nimotuzumab	Biological combination method	Epithelial tumours, carcinoma of head and neck, nasopharyngeal cancer	Asthma, rash, hypertension, microscopic haematuria, and fluctuations in blood pressure	[29,30,31]
Doxorubicin (DOX)	Double emulsion, hydrolysis	Breast cancer, ovarian cancer	Cardiac effects due to heart muscle failure	[32,33]
Ferumoxide and gadolinium	Hydrothermal reaction	MR imaging reticuloendothelial systems and liver stem cell labelling.	Can cause side effects such as severe backache when administered as intravenous bolus	[28,33,34,35,36,37,38]
Ferumoxsil (AMI-121)	Hydrothermal reaction	Bowel MRI, oral GI imaging, atherosclerosis, gastrointestinal as magnetic iron particle solution	Intestinal performance obstruction, metallic taste is a hindrance to routine use	[28,39]
Ferumoxtran (AMI-227)	Hydrothermal reaction	Noncancerous lymphatic tissue imaging	Coated with dextran, which can induce allergic reactions	[28,40,41,42]
Feruglose	Hydrothermal reaction	Blood pool agent, lymph nodes, and liver spleen imaging	Interferes with iron metabolism after biodegradation	[33,43]
Nanoworms (NW)	Co-precipitation	Tumour targeting, COVID-19	Manufacturing and disposal have negative impacts on the environment, potential side effects to the immune response are not fully explored	[44,45,46]
Resosvist (SHU555C)	Biological combination method	MRI signal intensity enhancement	Loss of signal in the healthy tissues	[47,48,49]
Hafnium oxide nanoparticle	Hydrothermal	Radiography, optical, sensing, oral cancer detection, biosensing, and electronic fields	Slow kinetics, low adsorption capacity, and leaching problem in low pH environments	[50,51,52,53,54,55]

**Table 2:** Summary of suitable NPs applications and limitations.

Type of NPs	Applications	Limitations	References
Carbon-based	Delivery of therapeutics Biomedical imaging Biosensors Tissue engineering Cancer therapy Catalysts Hydrogen storage systems Remediation of pollutants	Possibility of limited gas absorption and non-linear sensor response. Low reactivity of carbon Possibility of adverse effects on environment and health, if used in pure powders. Material agglomeration Limited detection range	[132,133,134,135,136,137,138]
Organic	Bioimaging (PA, PET, fluorescence) Drug delivery (PDT, PTT, and gene) therapies. Anticancer drugs Time released medications	Poor drug loading efficiency. Imbalances of pH at delivery sites. Poor recovery	[139,140,141,142,143,144,145]
Inorganic	Biomedical include things such as anti-cancer, bacterial, oxidant, inflammatory, diabetic and drug delivery, and bio-imaging. Agriculture includes nano-biosensors, herbicides, fertilizers, pesticides, and detection of pathogens, improving soil texture and precision farming. Industrial applications include purification of water, nanoencapsulation, nanoemulsions, and nanocomposites. Environmental applications include UV protection, contaminant sensors, biodegradable polymers, fuel cell catalysts, and pollutant scavengers.	Oxidative stress and inflammation in various organ systems Toxicity and vulnerability to oxidation Limited bioavailability Effective surface coatings with optimum performance Hydrophobic surface chemistry, merely soluble in non-polar solvents	[27,146,147,148,149,150,151,152,153]

**Table 3:** Therapeutic strategy outcome on treatment applications by various NP-based drugs.

Studied NPs	Type of Treatment	Therapeutic Strategy	Outcome	References
Dendrimers	Lung cancer/ imaging diagnostics	Biomarkers testing	Lowers the dose needed to produce images, increasing radioisotope efficiency.	[164]
Nanostructured transdermal hormone replacement	Menopausal symptoms	Hormone therapy	Improvements in climacteric symptoms. Every woman’s result from a bilateral mammography evaluation of breasts were normal.	[165]
Biocompatible gold–silica nanoshells	Prostate tumours	Photothermal ablation of prostate tumours	Effective GSN-mediated laser ablation, no significant changes in comparison to the “International Prostate Symptom Score or the Sexual Health Inventory for Men”.	[166,167]
Anti-CD19-Chimeric-Antigen-Receptor-Transduced T-Cells	B-Cell Malignancies	Stem cell transplant	Study is ongoing and is in follow-up stage currently. NCT01087294	[168]
Dihydroartemisinin-loaded magnetic nanoparticles	Breast cancer cells	Chemodynamic therapy	Proves to be more efficient and cost friendly, although more evaluations need to be conducted.	[169]
Belzutifan	Brain cancer	Targeted therapy	Only currently approved inhibitor, with more evaluation research ongoing.	[170]
Superparamagnetic iron oxide NPs	Glioblastomas (GBMs)	Magnetic hyperthermia	Long-term stabilization achieved for phase-I trial stage.	[171]

## Data Availability

Not applicable.
